# Associations between Cannabis Consumption Methods and Cannabis Risk Perception

**DOI:** 10.3390/ijerph21080986

**Published:** 2024-07-27

**Authors:** Namkee G. Choi, C. Nathan Marti, Bryan Y. Choi

**Affiliations:** 1 Steve Hicks School of Social Work, University of Texas at Austin, Austin, TX 78712, USA; nate.marti@utexas.edu; 2 Department of Emergency Medicine, Philadelphia College of Osteopathic Medicine and BayHealth, Dover, DL 19901, USA; bryan_choi@bayhealth.org

**Keywords:** cannabis, risk perception, consumption mode, eating/drinking, vaping, dabbing

## Abstract

Given diversified cannabis products, we examined associations between cannabis consumption methods and cannabis risk perception of smoking cannabis 1–2 times a week. Using the 2022 U.S. National Survey on Drug Use and Health data (N = 12,796 past-year adult cannabis users; M = 6127 and F = 6669), we used multinomial and binary logistic regression models. Smoking was the most prevalent method, followed by eating/drinking, vaping, and dabbing. One-half of cannabis users reported no perceived risk of smoking cannabis 1–2 times a week, 37.5% perceived slight risk, 9.2% moderate risk, and 2.9% great risk. Those with moderate or great risk perception had a lower likelihood of using 4+ methods of consumption (e.g., RRR = 0.40, 95% CI = 0.20, 0.77 for great risk perception). Any perceived risk was associated with higher odds of edibles/drinks only (e.g., aOR = 2.81, 95% CI = 1.43, 5.54 for great risk perception). Along with medical use and CUD, sociodemographic factors, mental illness, and other substance use were also significant correlates of cannabis consumption methods. Understanding the varying risk perceptions associated with different consumption methods is needed for harm reduction initiatives. More research is needed on cannabis products, particularly edibles/drinks and dabs/concentrates, to better understand the potential risks associated with them.

## 1. Introduction

As of 13 May 2024, 24 states and the District of Columbia in the United States (U.S.) fully legalized cannabis, 13 other states legalized medical use, and a total of 32 states decriminalized its use [1]. With increasing numbers of U.S. states where cannabis use is fully or partially legal over the past decade, the perception of harms associated with regular cannabis use has been declining [2,3,4,5]. The global movement toward legalization of cannabis has also contributed to increasing public perception that cannabis is safe [6]. Data from the 2002–2019 U.S. National Study on Drug Use and Health (NSDUH), including over a million U.S. residents age 12+, showed that in contrast to the perceived risk of having 5+ drinks 1–2 times a week that largely stayed stable among all age groups, the perceived risk of smoking cannabis 1–2 times a week became more permissive with time across ages, with the 18–34 age group showing the largest decrease in risk perceptions [7].

With expanding cannabis legalization and decreasing risk perceptions, the availability of and access to a variety of cannabis products with varying Δ^9^-tetrahydrocannabinol (THC) potency have been increasing [8,9,10]. As diverse cannabis products have become easily accessible at cannabis dispensaries, via online stores, and other commercial venues, cannabis users can choose different methods of consumption and use a combination of methods [11,12]. While smoking is still the most prevalent consumption method, vaping, dabbing, and topical use have been prevalent routes of administration among young people and adults of all ages [8,13,14,15]. The prevalence of edibles, drinks, and other oral forms of cannabis administration has also increased significantly among all age groups, but especially among older users [16]. Oils and oral solutions are most frequently used among cancer patients [17]. A study of young adult cannabis users, both medical and nonmedical, also found high rates of concentrate and edible use, especially among those who also use illicit drugs and misuse prescription drugs [18].

Different modes of consumption carry different risks of harm. Smoking cannabis is associated with various adverse respiratory system outcomes (e.g., bronchitis, lung function) and may increase the risk of suicidality in young individuals [19,20]. Vaporizing cannabis and ingesting edibles appear to reduce respiratory system problems, but may come with other risks (e.g., delayed peak plasma concentrations and impairment) [20,21,22,23,24,25].

Studies show that cannabis users tend to understand the relative risks associated with different consumption methods; however, their understanding may not always be accurate. For example, a study in Canada found that emerging adults perceived more risks from smoking and vaping marijuana than ingesting edibles, from higher-potency THC-dominant strains than lower-potency CBD-dominant strains, and from simultaneous use of cannabis and tobacco than using cannabis alone or simultaneous use of cannabis and alcohol [26]. A study of U.S. young adults also found that from 2016 to 2018, perceptions of no harm from edibles increased but declined for smoking cannabis [27].

Research has also shown that perceptions of cannabis risk are also strongly associated with cannabis use frequency. Risk perception is lower among frequent (e.g., daily) users, whereas a greater perceived risk of harm is associated with decreased cannabis use frequency [5,28,29,30,31]. However, a study of young adult cannabis users found that more cannabis problems were associated with perceiving more risk and poorer mental health [32]. An NSDUH-based study of those age 50 and older also found that among past-year users, those who had cannabis use disorder (CUD) were 3.5 times more likely than those without the disorder to report moderate/great risk perception about smoking cannabis [33]. These and other studies suggest that cannabis users understand the negative impact of problematic use on their physical and mental health [34,35,36,37].

Cannabis risk perceptions were also found to be associated with sociodemographic factors and cannabis use reasons. For example, higher education and income and medicinal cannabis use were found to be associated with lower risk perceptions, while female sex and non-white race/ethnicity were found to be associated with higher risk perceptions [33,38,39,40]. Lower risk perceptions among medicinal users are expected, as their motivation for cannabis use is to seek relief from physical health (e.g., chronic pain) and/or mental health problems [4,34,41,42,43].

Despite the diversification of cannabis products and consumption modes, little research has been conducted on the associations between cannabis consumption methods and cannabis risk perception among nationally representative samples. Cannabis risk perception may be a factor in some cannabis users’ choice of cannabis consumption methods. In this study, based on the 2022 U.S. NSDUH that included questions/responses about cannabis use methods for the first time in the NSDUH history, we examined the associations of the number and type of cannabis consumption methods with cannabis risk perceptions among adult (i.e., age 18+) cannabis users. The study hypotheses were: (H1) the likelihood of using 4+ methods would be lower among those with moderate or great risk perception; and (H2) the likelihood of the consumption of edibles and drinks only would be higher among those with moderate or great risk perception, controlling for sociodemographic variables, physical and mental health statuses, other substance use, medicinal cannabis use, cannabis use frequency, and CUD. The findings will provide valuable insights into cannabis users’ consumption modes and their association with cannabis risk perception.

## 2. Materials and Methods

### 2.1. Data Source

The NSDUH data are collected annually by the Substance Abuse and Mental Health Services Administration from the U.S. civilian, non-institutionalized, aged 12+ population to measure the prevalence of substance use, mental and substance use disorders, behavioral health treatment, and physical/functional health and healthcare use. The NSDUH uses a multi-stage, stratified sampling design, with states as the first level of stratification, and an independent, multistage area probability sample within each state and the District of Columbia [44]. In the aftermath of the COVID-19 pandemic, NSDUH data collection in 2022 was conducted using both in-person and web-based modes, and respondents had a choice to complete the survey in-person or via the web. The 2022 NSDUH public use data set includes responses from a total of 59,069 individuals who completed an in-person or web-based NSDUH survey. Of these, we focused on 12,796 adults (age 18+, representing over 58.8 million U.S. adults or 23% of the adult population) who reported cannabis use in the preceding 12 months. The analysis of de-identified public use data was exempt from the authors’ institutional review boards’ approval.

### 2.2. Measures

#### 2.2.1. Past-Year Cannabis Use Frequency, Medical Use, and Cannabis Use Disorder (CUD)

All NSDUH respondents were asked if they have ever (even once) used “marijuana or any cannabis product, sometimes called pot, weed, hashish, or concentrates, excluding CBD or hemp products”. For respondents who answered the ever-use question affirmatively, the first age of use and time since last use were queried. Past-year users were defined as those who used any cannabis in the preceding 12 months. Past year users were also asked about the number of days used in the past 12 months (1–11, 12–49, 50–99, 100–299, and 300–365) and any or all use recommended by a doctor.

CUD and CUD severity among past-year users were determined based on the *DSM-5* criteria [45]. The *DSM-5* defines CUD as a “problematic pattern of cannabis use leading to clinically significant impairment or distress” in at least 2 of 11 realms of functioning within a 12-month period: tolerance; withdrawal; unsuccessful efforts to cut down or control use; failure to fulfill obligations at work, school, or home; continued use despite recurrent physical or psychological consequences of use; and continued cannabis use despite having recurrent social or interpersonal problems caused or exacerbated by the effects of cannabis. The *DSM-5* CUD severity levels were mild (positive answers to 2–3 realms), moderate (positive answers to 4–5 realms), or severe (positive answers to 6+ realms).

#### 2.2.2. Cannabis Consumption Methods

Questions about the “ways” that cannabis users used “marijuana or any cannabis products” in the past 12 months (and the past 30 days) included: smoking (such as in joints, pipes, bongs, blunts, or hookahs); vaping (using vape pens, dab pens, tabletop vaporizers, or portable vaporizers); dabbing waxes, shatter, or concentrates (dabbing hereafter); eating or drinking; drops/strips/lozenges/sprays in mouth; applying as a lotion, cream, or a patch to skin; pills; and/or some other way (yes = 1, no = 0). The above methods were summed up to obtain the total number of methods used, resulting in values ranging from 0 to 8.

#### 2.2.3. Cannabis Risk Perception

All NSDUH respondents were asked a question, “*How much do people risk harming themselves physically and in other ways when they smoke marijuana once or twice a week*?” The response categories were no risk, slight risk, moderate risk, and great risk.

#### 2.2.4. Sociodemographic Factors

Sociodemographic factors were age (18–34, 35–49, 50–64, and 65+ years); gender; race/ethnicity; education (no college degree vs. college degree); and income (<poverty line, up to 2x poverty line, >2x poverty line).

#### 2.2.5. Physical Health Status, Past-Year Mental Health Status, and Other Substance Use

Physical health status was measured with the number (0–10) of diagnosed health conditions including asthma, cancer, COPD, diabetes, heart disease, hepatitis, HIV/AIDS, hypertension, kidney disease, and liver disease. Mental health status was measured with any past-year mental illness (none, mild, moderate, serious [any mental, behavioral, or emotional disorder—excluding developmental and substance use disorders] that substantially interfered with or limited one or more major life activities [46]). Other substance use problems were measured with nicotine dependence [47], *DSM-5* alcohol use disorder and any past-year prescription psychotherapeutics (including perception pain relievers, tranquilizers, and sedatives) disorder, and use of illicit drugs excluding cannabis.

### 2.3. Analysis

We used Stata/MP 18’s svy function (College Station, TX, USA) and subpop command in all analyses to account for the NSDUH’s multi-stage, stratified sampling estimates to ensure that variance estimates incorporated the full sampling design. All estimates presented in this study are weighted, except sample sizes. First, we presented sociodemographic and health characteristics, other substance use, and the perceived risk of smoking cannabis 2–3 times a week among past-year cannabis users. Second, we presented cannabis use characteristics, including the proportion of those who reported any medical use, cannabis use frequency, CUD severity, and consumption methods. Third, we used multinomial logistic regression analyses to test H1 (association between the number of cannabis consumption methods and cannabis risk perception). We used three categories for the number of methods as the dependent variable: a single (1) method (reference category), 2–3 methods, and 4+ methods. Covariates were the sociodemographic and health status variables, other substance use, and CUD. Due to the multicollinearity between cannabis use frequency and CUD based on variance inflation factor values (using a cut-off of 2.50 [48]), we only entered CUD in the model. Fourth, we fitted four binary logistic regression models to test H2 (associations between different modes of cannabis consumption and cannabis risk perception), with smoking only, edibles or drinks only, any vaping, and any dabbing as the dependent variables. “Any vaping” and “any dabbing” were chosen, as only small proportions of past-year cannabis users had only vaping or any dabbing. We excluded 107 past-year users who did not provide valid data on their risk perception from the multivariable analyses. Multinomial and binary logistic regression model results are presented as relative risk ratios (RRRs) and adjusted odds ratios (aOR), respectively, with 95% confidence intervals (CI). Significance was set at *p* < 0.05.

## 3. Results

### 3.1. Sociodemographic and Health Status and Cannabis Risk Perceptions of Past-Year Cannabis Users

Table 1 shows that nearly one-half of past-year cannabis users were age 18–34, a little more than a quarter were age 35–49, and nearly one-fifth were age 50–64. Nearly two-thirds were non-Hispanic whites, and almost 30% had a college education. Overall, they were physically healthy, but nearly 40% had any mental illness. Nearly one-sixth had nicotine dependence, about a quarter had alcohol use disorder, and a little more than a quarter reported having used illicit drugs. One-half of past-year users perceived no risk of smoking cannabis 1–2 times a week, almost 38% perceived a slight risk, 9% perceived a moderate risk, and 3% perceived a great risk.

### 3.2. Cannabis Use Characteristics and Consumption Methods among Past-Year Cannabis Users

Table 2 shows that of all users, 17% reported any medical use, nearly one-half used on 100–365 days, and 30% had CUD. On average, past-year cannabis users used two different modes of cannabis consumption. Smoking was the most prevalent method, with almost 80% having smoked cannabis in the past year, but only 30% of all users used smoking alone. Eating or drinking, with 47% reporting consumption of edibles/drinks, was the second most prevalent method, but less than 10% reported using them alone. More than one-third of all users reported vaping and a little more than one-sixth reported dabbing; however, only mall proportions reported using these methods alone (3.4% for vaping alone and 0.7% for dabbing alone). Topical use and drops/strips/lozenges/sprays in the mouth were used by less than 10% of past-year users, and unspecified other methods were used by about one-sixth of all users.

**Figure 1:**
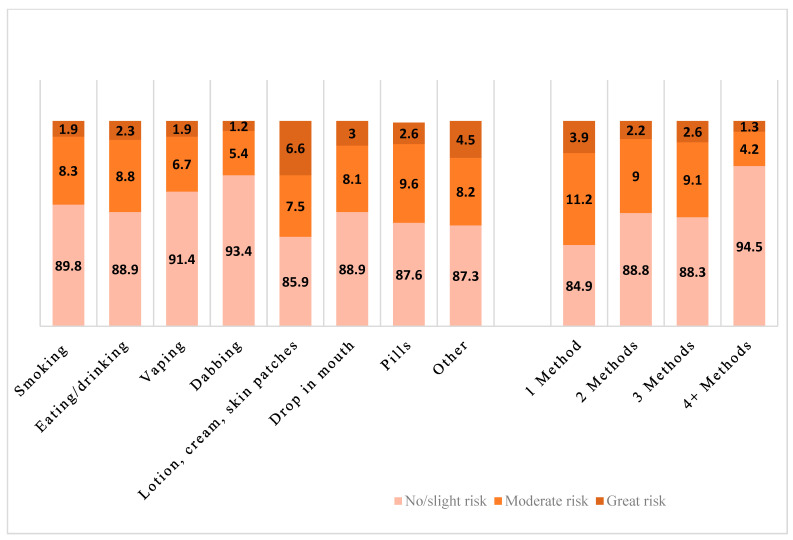
Cannabis risk perception by consumption method among past-year cannabis users.

Figure 1 shows risk perceptions by each consumption method and by the number of methods used. Those who used any vaping and any dabbing had a more permissive risk perception than the users who used other methods (e.g., vaping; F[1.90, 94.87] = 8.36, *p* < 0.001). It also shows that the users who used four or more methods had a more permissive perception, especially compared to the users who used only one method (F[4.86, 242.76] = 5.82, *p* < 0.001).

Further analyses also showed that those who used dabbing had the highest frequency of any cannabis use, with 53.4% having used daily or near daily (F[3.37, 168.33] = 100.49, *p* < 0.001) and the highest rate of a CUD (62.7% with any CUD and 13.2% with a severe CUD) (F[2.49, 124.37] = 133.59, *p* < 0.001). Those who used vaping also had a high rate of CUD (45.5% with any CUD and 8.1% with a severe CUD) (F[2.76, 137.87] = 82.16, *p* < 0.001). Compared to 18.9% of users who used only one method, 48.2% users who used 4+ methods used cannabis on 300+ days (F[8.24, 411.82] = 26.58, *p* < 0.001).

### 3.3. Associations between the Number of Cannabis Consumption Methods and Cannabis Risk Perception: Multinomial Logistic Regression Results

Table 3 shows that compared to those with no perceived risk, those with moderate (RRR = 0.41, 95% CI = 0.25, 0.67) and great risk perception (RRR = 0.40, 95% CI = 0.20, 0.77) were less likely to have used 4+ methods of consumption. Of the covariates, 35+ age groups, non-Hispanic Blacks and Hispanics, income up to 2x poverty line (compared to income >2x poverty line), and nicotine dependence were associated with a lower likelihood of using 2–3 or 4+ methods. On the other hand, higher numbers of medical conditions, any medical use, any severity of CUD, and illicit drug use were associated with a higher likelihood of using 2–3 or 4+ methods. Additionally, having a college degree was associated with a higher likelihood of using 2–3 methods, and moderate/severe mental illness was associated with a higher likelihood of using 4+ methods.

### 3.4. Associations between the Modes of Consumption and Cannabis Risk Perception: Binary Logistic Regression Results

Table 4 shows that slight, moderate, and great risk perceptions were associated with higher odds of consuming edibles/drinks only (aOR = 1.36, 95% CI = 1.01–1.80 for slight risk; aOR = 1.70, 95% CI = 1.14–2.51 for moderate risk; aOR = 2.81, 95% CI = 1.43–5.54 for great risk), and moderate risk perception was associated with lower odds of any vaping (aOR = 0.71, 95% CI = 0.53–0.95). Risk perceptions were not significantly associated with other modes of consumption.

Of the covariates, compared to the 18–34 age group, the older age groups had higher odds of smoking only or using edibles/drinks but lower odds of any vaping or dabbing. Women had lower odds of smoking only or any dabbing, but higher odds of using edibles/drinks only. Compared to non-Hispanic Whites, Blacks and Hispanics are more likely to use smoking only and less likely to use vaping or dabbing. College degree was associated with lower odds of smoking only or dabbing but higher odds of using edibles/drinks. Compared to those with higher income (>2x poverty), those with income below or <2x poverty had higher odds of smoking only; those with <2x poverty also had lower odds of using edibles/drinks only or any vaping; and those in poverty had higher odds of any dabbing. Those with moderate/severe mental illness had lower odds of smoking only and edibles/drinks only but higher odds of any vaping or dabbing. Those with any CUD had lower odds of smoking only and edibles/drinks only. Nicotine dependence was associated with higher odds of smoking only but lower odds of edibles/drinks only and any vaping. Alcohol use disorder was associated with higher odds of smoking only. Illicit drug use was associated with lower odds of smoking only but higher odds of any vaping or dabbing.

## 4. Discussion

In this study, we used a nationally representative sample of adult cannabis users to examine the associations between cannabis risk perception and cannabis consumption methods. Only 12% of past-year cannabis users endorsed moderate/great risk of smoking cannabis 1–2 times a week, with less than 3% of endorsing a great risk. This indicates that an absolute majority of cannabis users did not consider cannabis to be a harmful substance. As hypothesized, risk perception was significantly associated with consumption methods, as users with moderate or great risk perception were less likely to have used 4+ modes of consumption. The significant inverse association between risk perception and use of 4+ methods of consumption indicates that users with no/low risk perception will adopt multiple consumption methods, thus potentially exposing them to methods with varying degrees of potential harms. Users with slight, moderate, or great risk perception were also more likely to have used eating/drinking methods only, with the highest odds among those with great risk perception. The association between any perceived risk and using eating/drinking only is likely because cannabis users tend to believe that smoking is harmful but edible consumption is not [26]; however, some users are aware of delayed effects, unexpected highs, the unpredictability of the high, and inconsistency of THC distribution in edible products [49].

Our findings also show significant associations of cannabis consumption methods with CUD. It is not surprising that those with any CUD had higher likelihood of using multiple consumption methods and using dabbing. The odds of dabbing were more than four times higher among those with moderate or severe CUD compared to those without a CUD. While it is not possible to discern the order of using certain consumption methods and CUD with cross-sectional data, it is likely that cannabis use leads to lower risk perception, which then can lead to more frequent use and/or using high-potency cannabis products, resulting in addiction and CUD. Longitudinal studies of adolescents showed that while higher cannabis risk perceptions predicted lower cannabis use 2–4 years later, the negative association between past-year cannabis use and subsequent risk perception was stronger than that between risk perception and subsequent use [31,50]. A recent survey of Canadian and U.S. adults also found that cannabis users intend to increase their usage, with edibles attracting a rising level of interest from consumers [51]. With increasingly permissive legal environments for cannabis use and multiplication of cannabis products, CUD prevalence may increase. A study of nine studies published between 2016 and 2022 found that an increase in adult CUD prevalence was associated with recreational cannabis legalization [52].

The proportions of daily/near daily users and those with a CUD among dabbing users are especially concerning. Along with the high THC concentrations, many risks with dabs or cannabis concentrates have been reported, including agitation, neurotoxicity, cardiotoxicity, end-organ damage, and considerable residual solvent and pesticide contamination [53,54,55,56]. The high THC concentration of dabs is also likely to be addictive, posing increased risk for high-frequency use and CUD. A systematic review found that use of higher potency cannabis, relative to lower potency cannabis, was associated with an increased risk of psychosis and CUD [57].

Our findings also show that medical rather than non-medial cannabis users use more methods, including dabbing. Research has shown that cannabis users in cannabis-legal states (medical or recreational), compared to those in states where cannabis is not legal, were significantly more likely to use highly potent cannabis concentrates [58]. This suggest that easy access to and availability of a variety of cannabis products likely facilitated the use of high-potency products.

The associations of cannabis consumption methods with sociodemographic variables, mental health statuses, and other substance use were also very informative. Middle-aged and older adults and minoritized groups tended to use fewer methods overall, but those with mental illness and those who used illicit drugs used more methods. Our findings also show that smoking only or edibles/drinks only were used among older users, but vaping and dabbing were used among the 18–34 age group. Further research is needed to examine sex and racial/ethnic differences in cannabis consumption methods; however, our findings suggest that age, gender, and cultural norms likely play a role in choosing cannabis consumption methods. The significant associations of vaping and dabbing with mental illness and illicit drug use indicate serious problems among those, especially young adults, who resort to these modes of cannabis consumption and the need for them to access mental health and substance use treatment.

This study has a few limitations. First, since the NSDUH is based on self-reported data, the validity of respondents’ self-reported behaviors and other variables was not ascertained. Reporting of cannabis and other substance use may have been affected by social desirability bias. Second, the NSDUH’s cannabis risk perception data are limited to smoking cannabis. Cannabis users may have different risk perceptions of different use methods. Third, while the overall sample size is big, the number of cannabis users who used lotions, creams, skin patches, mouth drops, and pills was still small for meaningful analysis for these methods of consumption. Fourth, other than the medical use variable, data on specific conditions for medical use were not available. This limited a more in-depth analysis of consumption modes and intended effects. Fifth, only correlation, not causation, can be inferred from cross-sectional data.

## 5. Conclusions and Implications

This study shows that cannabis consumption modes are significantly associated with cannabis risk perception. The findings have significant public health implications, as growing shares of the U.S. population use cannabis. First, more attention needs to be paid to assessing modes of consumption and their respective health effects to guide public education and individual and public health intervention strategies. In particular, public health efforts could focus on educating consumers about the delayed onset and longer duration of effects associated with edibles and drinks. Providing guidance on proper dosing and consumption techniques could help reduce the risk of adverse reactions. Regulatory measures could be implemented to ensure accurate labeling of edibles and drinks, including information on potency and recommended serving sizes, to mitigate potential risks associated with overconsumption. Second, public health interventions also need to focus on harm reduction strategies for individuals who use vaping and dabbing, given their high frequency use and high CUD rates. This may include promoting safer consumption practices, such as using appropriate equipment and avoiding high-potency products, along with continued monitoring and research into the health effects of vaping, dabbing, and concentrate use. Third, more research is needed to better understand different cannabis products like lotions, pills, mouth drops, and others and to develop targeted public health interventions aimed at promoting safer cannabis use practices and minimizing potential harms associated with different consumption methods. Fourth, continued research is also needed to explore the underlying factors driving differences in risk perception among cannabis users based on consumption methods. Longitudinal studies could provide insights into how risk perceptions evolve over time and their impact on consumption patterns and related physical and mental health outcomes.

## Figures and Tables

**Table 1:** Sociodemographic and health characteristics and cannabis risk perception of past-year adult cannabis users (N = 12,796).

		%/Mean (SE)
Age (years, %)		
	18–34	47.3
	35–49	26.0
	50–64	19.0
	65+	7.7
Male (%)		54.9
Race/ethnicity 9%)		
	Non-Hispanic White	64.1
	Non-Hispanic Black	12.7
	Hispanic	16.1
	Other	7.1
College graduate (%)		29.8
Income (%)		
	Below poverty line	16.6
	Up to 2x poverty line	20.3
	More than 2x poverty line	63.2
Number of medical conditions, M (SE)		0.54 (0.02)
Past-year any mental illness (%)		
	None	60.6
	Mild	14.7
	Moderate/severe	14.7
Past-year nicotine dependence (%)		14.6
Past-year alcohol use disorder (%)		24.9
Past-year psychotherapeutics use disorder (%)		5.8
Past-year any illicit drug use excluding cannabis (%)		26.8
Perceived risk of smoking 1–2 times a week (%)		
	No risk	49.9
	Slight risk	37.5
	Moderate risk	9.2
	Great risk	2.9
	Missing	0.5

**Table 2:** Cannabis use characteristics among past-year cannabis users (N = 12,796).

			%/Mean (SE)
Any medical use (%)			16.9
Days cannabis was used (%)			
	1–11		24.7
	12–49		16.2
	50–99		9.9
	100–299		23.9
	300+		25.3
Any past-year cannabis use disorder (%)			30.4
	Mild		16.9
	Moderate		8.4
	Severe		5.1
Average no. of methods of cannabis consumption, M (SE)			2.13 (0.03)
Consumption methods in group (%)			
	1 method		44.0
	2 methods		24.5
	3 methods		16.2
	4+ methods		15.3
Specific method of consumption (%)			
	Smoking		78.6
		Smoking only	30.6
	Eating or drinking		47.3
		Eating or drinking only	9.2
	Vaping		35.7
		Vaping only	3.4
	Dabbing waxes, shatter, or concentrates		17.4
		Dabbing waxes, shatter, or concentrations alone	0.7
	Lotion, cream, or patches to skin		8.9
	Drops, strips, lozenges, or sprays in mouth		7.3
	Pills		2.6
	Other methods		15.3

**Table 3:** Associations of the number of cannabis consumption methods with cannabis risk perception: results from multinomial logistic regression.

		Compared to Using Only One Method	
		2–3 Methods RRR (95% CI)	4+ Methods RRR (95% CI)
Risks of smoking cannabis 1–2 times a week: vs. No risk			
	Slight risk	0.96 (0.79–1.18)	0.83 (0.65–1.04)
	Moderate risk	0.82 (0.63–1.06)	0.41 (0.25–0.67) **
	Great risk	0.66 (0.38–1.15)	0.40 (0.20–0.77) **
Age: vs. 18–34 years			
	35–49 years	0.78 (0.66–0.93) **	0.77 (0.62–0.96) *
	50–64 years	0.53 (0.43–0.66) ***	0.33 (0.22–0.50) ***
	65+ years	0.43 (0.30–0.61) ***	0.34 (0.21–0.54) ***
Female vs. Male		1.11 (0.96–1.28)	1.17 (0.93–1.46)
Race/ethnicity: vs. Non-Hispanic White			
	Non-Hispanic Black	0.57 (0.43–0.75) ***	0.27 (0.19–0.38) ***
	Hispanic	0.79 (0.63–0.99) *	0.61 (0.46–0.82) **
	Other	1.04 (0.77–1.40)	1.00 (0.74–1.36)
College degree vs. no college degree		1.28 (1.07–1.53) **	1.11 (0.87–1.42)
Income: vs. More than 2x poverty line			
	Below poverty line	0.80 (0.64–1.00)	0.78 (0.60–1.00)
	Up to 2x poverty line	0.81 (0.67–0.98) *	0.79 (0.63–0.99) *
Number of medical conditions		1.15 (1.04–1.28) **	1.33 (1.17–1.51) ***
Past-year any mental illness: vs. None			
	Mild	1.15 (0.91–1.44)	1.09 (0.80–1.49)
	Moderate/severe	1.19 (0.98–1.44)	1.51 (1.13–2.02) **
Any medicinal cannabis use: vs. no medicinal use		1.48 (1.20–1.82) ***	3.05 (2.33–3.99) ***
Cannabis use disorder severity: vs. no cannabis use disorder			
	Mild	1.81 (1.43–2.28) ***	3.26 (2.46–4.33) ***
	Moderate	1.99 (1.35–2.93) **	4.44 (3.04–6.50) ***
	Severe	1.82 (1.35–2.44) ***	5.66 (3.83–8.37) ***
Nicotine dependence		0.54 (0.44–0.67) ***	0.54 (0.40–0.74) ***
Alcohol use disorder		0.99 (0.82–1.20)	0.91 (0.72–1.16)
Any psychotherapeutics disorder		0.96 (0.58–1.58)	0.93 (0.64–1.35)
Illicit drug use excluding cannabis		1.47 (1.23–1.75) ***	2.03 (1.62–2.55) ***
Model statistics		N = 12,689; Population size: 58,499,557; Design df = 50; F (48,3) = 35.46; *p* = 0.007	

* *p* < 0.05; ** *p* < 0.01; *** *p* < 0.001.

**Table 4:** Associations of the mode of cannabis consumption with cannabis risk perception: results from logistic regression.

		Smoking Only	Eating/Drinking Only	Any Vaping	Any Dabbing
		aOR (95% CI)	aOR (95% CI)	aOR (95% CI)	aOR (95% CI)
Risks of smoking cannabis 1–2 times a week: vs. No risk					
	Slight risk	0.91 (0.76–1.10)	1.36 (1.03–1.80) *	0.91 (0.77–1.07)	0.84 (0.66–1.06)
	Moderate risk	1.14 (0.86–1.52)	1.70 (1.14–2.53) **	0.71 (0.53–0.95) *	0.68 (0.44–1.06)
	Great risk	0.86 (0.56–1.30)	2.81 (1.43–5.54) **	0.69 (0.39–1.24)	0.49 (0.23–1.05)
Age: vs. 18–34 years					
	35–49 years	1.30 (1.04–1.62) *	1.41 (1.08–1.84) *	0.65 (0.56–0.76) ***	0.53 (0.44–0.65)
	50–64 years	2.28 (1.81–2.87) ***	1.28 (0.87–1.90)	0.32 (0.24–0.42) ***	0.31 (0.20–0.48)***
	65+ years	2.36 (1.70–3.27) ***	1.32 (0.82–2.13)	0.32 (0.22–20.48) ***	0.20 (0.09–0.47)***
Female vs. Male		0.78 (0.64–0.94)*	1.55 (1.19–1.99) **	0.96 (0.83–1.10)	0.74 (0.59–0.93) *
Race/ethnicity: vs. Non-Hispanic White					
	Non-Hispanic Black	2.55 (1.99–3.27) ***	0.75 (0.49–1.17)	0.42 (0.32–0.54) ***	0.32 (0.24–0.41) ***
	Hispanic	1.51 (1.23–1.85) ***	0.80 (0.50–1.30)	0.79 (0.65–0.97) *	0.64 (0.49–0.83) ***
	Other	1.05 (0.80–1.39)	0.91 (0.62–1.35)	0.80 (0.61–1.05)	0.79 (0.58–1.07)
College degree vs. No college degree		0.51 (0.42–0.63) ***	2.28 (1.74–2.99) ***	1.01 (0.89–1.16)	0.49 (0.38–0.62) ***
Income: vs. More than 2x poverty line					
	Below poverty line	1.45 (1.20–1.76) ***	0.71 (0.45–1.11)	0.85 (0.68–1.08)	1.27 (1.03–1.57) *
	Up to 2x poverty line	1.60 (1.30–1.98) ***	0.55 (0.38–0.80) **	0.83 (0.69–0.99) *	1.03 (0.84–1.27)
Number of medical conditions		0.81 (0.70–0.94)**	1.09 (0.93–1.27)	1.11 (0.98–1.25)	1.02 (0.92–1.12)
Past-year any mental illness: vs. None					
	Mild	0.82 (0.66–1.03)	1.29 (0.90–1.86)	1.02 (0.86–1.21)	1.08 (0.82–1.43)
	Moderate/severe	0.64 (0.52–0.80) ***	1.41 (1.04–1.92) *	1.18 (0.98–1.43)	1.21 (0.91–1.61)
Any medicinal cannabis use: vs. no medicinal use		0.63 (0.50–0.79) ***	0.57 (9.34–0.93) *	1.43 (1.19–1.71) ***	1.85 (1.47–2.33) ***
Cannabis use disorder severity: vs. no cannabis use disorder					
	Mild	0.72 (0.58–0.89) **	0.14 (0.05–0.36) **	2.26 (1.93–2.65) ***	3.17 (2.51–4.00) ***
	Moderate	0.63 (0.45–0.89) *	0.08 (0.03–0.18) ***	2.54 (1.90–3.39) ***	4.23 (3.09–5.79) ***
	Severe	0.60 (0.42–0.86) **	0.11 (0.03–0.38) **	2.45 (1.84–3.28) ***	4.86 (3.42–6.89) ***
Nicotine dependence		2.30 (1.80–2.93) ***	0.42 (0.25–0.72) **	0.59 (0.49–0.72) ***	0.97 (0.75–1.25)
Alcohol use disorder		1.20 (1.04–1.38) *	0.76 (0.54–1.06)	1.00 ((0.85–1.17)	0.79 (0.65–0.97) *
Any psychotherapeutics disorder		0.92 (0.57–1.46)	1.70 (0.81–3.57)	1.03 (0.75–1.42)	1.09 (0.71–1.68)
Illicit drug use excluding cannabis		0.63 (0.52–0.76) ***	0.74 (0.50–1.09)	1.77 (1.48–2.11) ***	2.15 (1.73–2.68) ***
Model statistics		N = 12,689; Design df = 50; F (24,27) = 14.67; *p* < 0.001	N = 12,689; Design df = 50; F (24,27) = 12.56; *p* < 0.001	N = 12,689; Design df = 50; F (24,27) = 18.95; *p* < 0.001	N = 12,689; Design df = 50; F (24,27) = 36.23; *p* < 0.001

* *p* < 0.05; ** *p* < 0.01; *** *p* < 0.001.

## Data Availability

This study is based on de-identified public-domain data (The National Survey on Drug Use and Health).
